# Genome-Wide Association Study Reveals Genetic Link between Diarrhea-Associated Entamoeba histolytica Infection and Inflammatory Bowel Disease

**DOI:** 10.1128/mBio.01668-18

**Published:** 2018-09-18

**Authors:** Genevieve L. Wojcik, Chelsea Marie, Mayuresh M. Abhyankar, Nobuya Yoshida, Koji Watanabe, Alexander J. Mentzer, Tommy Carstensen, Josyf Mychaleckyj, Beth D. Kirkpatrick, Stephen S. Rich, Patrick Concannon, Rashidul Haque, George C. Tsokos, William A. Petri, Priya Duggal

**Affiliations:** a Department of Genetics, Stanford University School of Medicine, Stanford, California, USA; b Division of Infectious Diseases and International Health, Department of Medicine, University of Virginia School of Medicine, Charlottesville, Virginia, USA; c Department of Medicine, Beth Israel Deaconess Medical Center, Harvard Medical School, Boston, Massachusetts, USA; d Nuffield Department of Medicine, Wellcome Trust Centre for Human Genetics, University of Oxford, Oxford, United Kingdom; e Wellcome Trust Sanger Institute, Genome Campus, Oxford, United Kingdom; f Department of Medicine, Cambridge University, Cambridge, United Kingdom; g Department of Public Health Sciences, Center for Public Health Genomics, University of Virginia School of Medicine, Charlottesville, Virginia, USA; h Vaccine Testing Center, University of Vermont College of Medicine, Burlington, Vermont, USA; i Genetics Institute and Department of Pathology, Immunology and Laboratory Medicine, University of Florida, Gainesville, Florida, USA; j International Centre for Diarrheal Disease Research, Dhaka, Bangladesh; k Department of Epidemiology, Johns Hopkins Bloomberg School of Public Health, Baltimore, Maryland, USA; Harvard T. H. Chan School of Public Health

**Keywords:** diarrhea, epidemiology, genomics, infectious disease, protozoa, public health

## Abstract

Entamoeba histolytica is the etiologic agent of amebic dysentery, though clinical manifestation of infection is highly variable ranging from subclinical colonization to invasive disease. We hypothesize that host genetics contribute to the variable outcomes of E. histolytica infection; thus, we conducted a genome-wide association study (GWAS) in two independent birth cohorts of Bangladeshi infants monitored for susceptibility to E. histolytica disease in the first year of life. Children with at least one diarrheal episode positive for E. histolytica (cases) were compared to children with no detectable E. histolytica infection in the same time frame (controls). Meta-analyses under a fixed-effect inverse variance weighting model identified multiple variants in a region of chromosome 10 containing loci associated with symptomatic E. histolytica infection. An intergenic insertion between *CREM* and *CCNY* (rs58000832) achieved genome-wide significance (*P* value from meta-analysis [*P*_meta_] = 6.05 × 10^−9^), and each additional risk allele of rs58000832 conferred 2.42 increased odds of a diarrhea-associated E. histolytica infection. The most strongly associated single nucleotide polymorphism (SNP) within a gene was in an intron of *CREM* (rs58468612; *P*_meta_ = 8.94 × 10^−8^), which has been implicated as a susceptibility locus for inflammatory bowel disease (IBD). Gene expression resources suggest associated loci are related to the lower expression of *CREM*. Increased *CREM* expression is also observed in early E. histolytica infection. Further, *CREM^−^*^/^*^−^* mice were more susceptible to E. histolytica amebic colitis. These genetic associations reinforce the pathological similarities observed in gut inflammation between E. histolytica infection and IBD.

## Abstract (precis)

Diarrhea is the second leading cause of death for children globally, causing 760,000 deaths each year in children less than 5 years old. Amebic dysentery contributes significantly to this burden, especially in developing countries. The identification of host factors that control or enable enteric pathogens has the potential to transform our understanding of disease predisposition, outcomes, and treatments. Our discovery of the transcriptional regulator cAMP-responsive element modulator (CREM) as a genetic modifier of susceptibility to amebic disease has implications for understanding the pathogenesis of other diarrheal infections. Further, emerging evidence for CREM in IBD susceptibility suggests that CREM is a critical regulator of enteric inflammation and may have broad therapeutic potential as a drug target across intestinal inflammatory diseases.

## INTRODUCTION

Despite drastic reductions in childhood mortality from 1990 to 2015, the Millennium Development Goal 4 failed to reach the target goal of two-thirds reduction in the mortality rate for children less than 5 years old (1). One of the leading causes of childhood mortality is diarrheal disease, leading to more than half a million deaths annually (2). The burden of diarrhea-related mortality and morbidity is disproportionately higher in developing countries. One endemic etiology of diarrheal disease in the developing world is amebiasis, an infection by the protozoan parasite Entamoeba histolytica (3). Observational studies have shown that preschool age children with a history of E. histolytica-associated diarrheal illness were more likely to be malnourished and stunted (4, 5). While the majority of infections by E. histolytica are asymptomatic (6), the 10% that develop disease can exhibit acute diarrhea, dysentery, amebic colitis, and amebic liver abscess (7).

Major risk factors for amebiasis include poor sanitation and hygiene, as transmission occurs via the ingestion of amebic cysts that are found in contaminated food and water (8). Within a group of 147 infants monitored for the first year of life in an urban slum of Dhaka, Bangladesh, 10.9% of children had at least one diarrheal episode positive for E. histolytica (9). Children were more likely to be infected with E. histolytica if they were born malnourished. These findings are consistent with prior evidence in preschool age children, which showed children with E. histolytica-associated diarrheal illness were more likely to be malnourished and stunted (5). By the end of 3 years of follow-up (from ages 2 to 5 years), 17% of children had at least one diarrheal episode positive for E. histolytica. Within a homogenous environment with presumed uniform exposure to the pathogen, it is not well understood why only a subset of individuals exposed exhibit infection, and subsequently why symptomatic disease develops only in a subset of those individuals.

One possible explanation for the observed heterogeneity in infection rates could be differences in host genetic susceptibility to infection and disease (10). In a study of preschool age children in Dhaka, Bangladesh, Duggal et al. identified a polymorphism in the leptin receptor, rs1137101 resulting in Q223R, that was associated with infection by E. histolytica compared to children without this polymorphism (11). Later work elucidated the mechanism of action of this allele; glutamine at this position led to a decrease in STAT-3-dependent gene expression, which in turn led to an increase in host cell apoptosis during E. histolytica infection (12, 13). An additional association was found between human leukocyte antigen (HLA) class II alleles and E. histolytica infection in Bangladeshi children, specifically DQB1*0601 and the haplotype containing the DQB1*0601/DRB1*1501 heterozygote (14). To comprehensively identify loci conferring risk for diarrhea-associated Entamoeba histolytica infection, we conducted a genome-wide association study (GWAS) by implementing a meta-analysis of two existing birth cohorts of children in Dhaka, Bangladesh: PROVIDE (Performance of Rotavirus and Oral Polio Vaccines in Developing Countries) Study (15) and the Dhaka Birth Cohort (DBC) (9).

(This article was submitted to an online preprint archive [16].)

## RESULTS

### Study population.

Within the well-characterized DBC and PROVIDE studies, children were monitored twice weekly for a possible diarrheal episode. When a mother reported diarrhea in her child, a fecal sample was tested for E. histolytica. Within the DBC study (9), 312 children were reported to have diarrhea; 60 of these children had at least one diarrheal episode positive for E. histolytica within the first year of life. A total of 252 controls had a stool sample collected within the first year of life (diarrheal or normal monthly), and they were not positive for E. histolytica. Within the PROVIDE study (15), 110 children had at least one diarrheal episode positive for E. histolytica, while 322 did not have any E. histolytica*-*positive samples within the first year of life. Diarrhea-associated E. histolytica infection had no association (*P  > *0.05) with height-for-age Z-score (HAZ) at 1 year of age, the number of days that the child was exclusively breastfed, or sex (Table 1). Results were consistent between the DBC and PROVIDE studies (*P* value of heterogeneity from Q statistic [*P*_het_] > 0.05).

**TABLE 1:** Association of *E*. *histolytica* diarrhea cases and controls with key covariates

Covariate	Value for covariate in DBC (*n* = 312)		*P*^*a*^	Value for covariate in PROVIDE (*n* = 432)		*P*	*P*_het_^*b*^
	Controls (*n* = 252)	Cases (*n* = 60)		Controls (*n* = 322)	Cases (*n* = 110)		
HAZ_12_^*c*^ (mean)	−2.02	−2.02	0.997	−1.45	−1.47	0.833	0.854
No. of days of exclusive breastfeeding (mean)	122.67	130.54	0.425	125.54	122.99	0.688	0.408
Sex (% female)	56.35	56.67	0.964	54.66	52.73	0.726	0.789

a The *P* values compare the values for controls and cases in the DBC and PROVIDE studies.

b *P*_het_, *P* value of heterogeneity from Q statistic.

c HAZ_12_, height-for-age Z-score at 12 months.

### GWAS meta-analysis identifies significant association in *CREM-CUL2* region.

**FIG 1:**
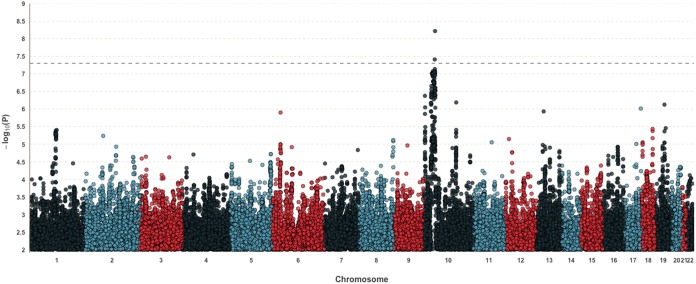
Manhattan plot of diarrhea-associated E. histolytica infection within the first year of life. The genome-wide significance threshold is indicated by a broken line at 5 × 10^−8^.

**FIG 2:**
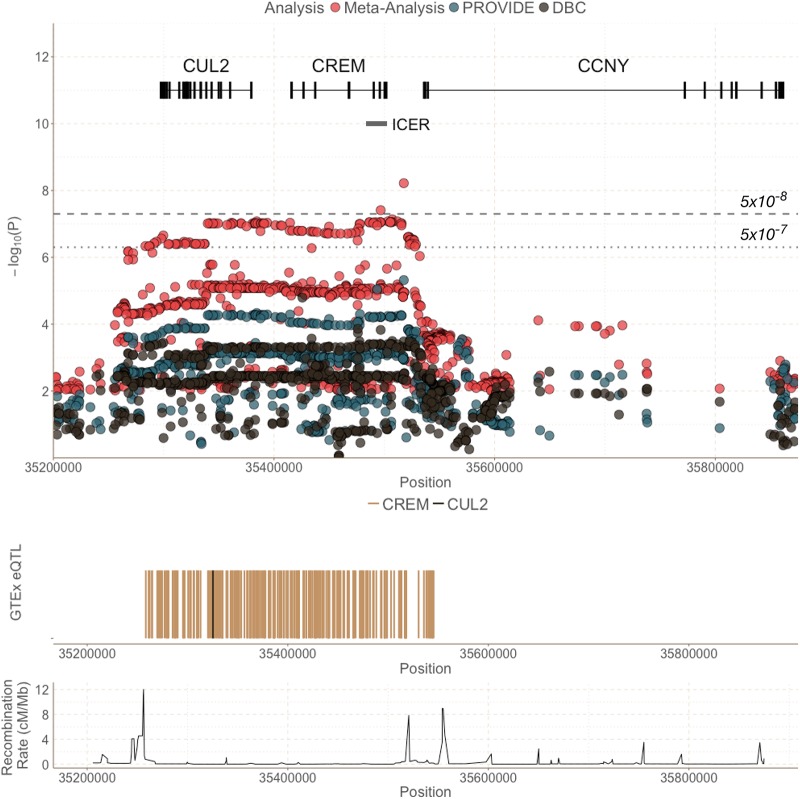
The *CUL2-CREM-CCNY* region and GTEx results of known expression quantitative trait loci (eQTL). The top graph shows both association results from individual studies (DBC [gray] and PROVIDE [blue]) as well as the meta-analysis of GWAS results (red) in this region. The middle graph shows GTEx eQTLs as vertical lines for GWAS variants with *P  < *0.01 within the meta-analysis. The vast majority of overlapping associated SNPs from the meta-analysis are eQTLs for *CREM*, indicating a role for *CREM*, instead of *CUL2* or *CCNY*. The bottom graph shows the recombination rate (centimorgans per megabase [cM/Mb]) for this region, illustrating the large block of linkage disequilibrium in this region.

A total of 6.7 million single nucleotide polymorphisms (SNPs) with a minor allele frequency (MAF) greater than 5% in both cohorts were analyzed for association with diarrhea-associated E. histolytica infection within the first year of life. A genome-wide association analysis was performed separately for each study (DBC and PROVIDE), in which logistic regression assuming an additive model was conducted on each SNP, followed by a fixed-effect meta-analysis across the two studies (Fig. 1 and Table 2). The first five principal components were included as covariates in all analyses to control for possible confounding due to population substructure. The top genetic association (rs58000832, *P*_meta_ = 6.05 × 10^−9^, MAF = 23.0%) was identified on chromosome 10 in a region covering three genes: *CUL2* (cullin 2), *CREM* (cAMP-responsive element modulator), and *CCNY* (cyclin Y) (Fig. 2). Individuals with at least one copy of the CA insertion at rs58000832, an intergenic insertion between *CREM* and *CCNY*, had 2.42 times increased odds of diarrhea-associated infection within the first year of life compared to individuals with no copies of this insertion. The most strongly associated SNP located in a gene (rs58468612, *P* value from meta-analysis [*P*_meta_] = 8.94 × 10^−8^) was in an intron of *CREM*. Within this region of association, there is a large linkage disequilibrium block in this region that underlies *CREM* and *CUL2*, flanked by two recombination peaks (Fig. 2). With this high level of correlation between the variants, it is not possible to pinpoint an individual causal SNP from the GWAS alone. An intronic SNP within *CUL2*, rs2148483, was directly genotyped in both cohorts and was consistent with the imputed variants (*P*_meta_ = 9.64 × 10^−8^, odds ratio from meta-analysis [OR_meta_] = 2.24, MAF  = 21.2%), ensuring the validity of analyses with imputed variants.

**TABLE 2:** Genome-wide significant associations from meta-analysis and stratified analyses

rsID^*a*^	Position	A0^*b*^	A1^*c*^	AF (%)^*d*^	DBC^*e*^			PROVIDE^*f*^			META^*g*^		*P*_het_^*h*^
					OR [95% CI]	*P*	INFO	OR [95% CI]	*P*	INFO	OR [95% CI]	*P*	
rs58000832	35517635	C	CA	23.04	2.39 [1.49, 3.85]	3.27 × 10^−4^	0.93	2.44 [1.67, 3.58]	4.80 × 10^−6^	0.94	2.45 [1.83, 3.29]	6.05 × 10^−9^	0.95
rs58994923	35496699	TA	T	21.18	2.56 [1.57, 4.15]	1.48 × 10^−4^	0.98	2.18 [1.49, 3.2]	6.14 × 10^−5^	0.99	2.36 [1.76, 3.18]	3.89 × 10^−8^	0.62
rs2148483*	35341301	G	A	21.24	2.31 [1.44, 3.71]	4.89 × 10^−4^	1.00	2.2 [1.5, 3.22]	5.36 × 10^−5^	1.00	2.24 [1.67, 3.02]	9.64 × 10^−8^	0.87

a rsID, rs identifier. The three SNPs are all on chromosome 10. The asterisk indicates the directly genotyped SNP with the strongest association.

b A0, risk allele.

c A1, non-risk allele.

d AF, allele frequency.

e OR, odds ratio; 95% CI, 95% confidence interval; *P*, *P* value; INFO, info score from IMPUTE2; DBC, Dhaka Birth Cohort.

f PROVIDE, PROVIDE Study.

g META, meta-analysis results.

h *P*_het_, *P* value of heterogeneity from Q statistic.

### *CREM/CUL2* haplotypes enriched for South Asian populations.

Due to the high levels of linkage disequilibrium in this area, we investigated possible associations with haplotypes, or the sequence of variants along a chromosome across this region, which may be inherited together and predispose the child to diarrhea-associated E. histolytica. There were 11 distinct haplotypes in the *CREM*/*CUL2* region within PROVIDE using 25 associated SNPs on chromosome 10 (physical positions, 35,273,439 to 35,513,323). The most associated haplotype (*P  =  *4.73 × 10^−4^) conferred 1.95 times increased odds of diarrhea-associated E. histolytica infection with each copy of the haplotype (see Table S1 at https://doi.org/10.6084/m9.figshare.7034456). This single haplotype encompasses both *CREM* and *CUL2*. When the haplotype was split into the two genic regions (*CREM* and *CUL2,* respectively), there was little difference in association results due to extensive linkage disequilibrium across the region (see Table S2 at https://doi.org/10.6084/m9.figshare.7034456).

We examined the frequencies of both SNPs and haplotypes in our study compared to publicly available data with the 1000 Genomes Project Phase 3 data (1000Genomes) in five main continental groups: Africa, the Americas, East Asia, South Asia, and Europe (17). SNP frequencies were consistent between our study and external reference data. We found results consistent between our studies and the Bangladeshi reference population (BEB) from 1000Genomes. The top associated genic SNP (rs58468612) had a MAF of 21.1% within our study and 22.1% within BEB. The risk haplotype identified in PROVIDE was found at the highest frequency in South Asian (19.2%) and European (17.0%) populations, exhibiting significant enrichment compared to other groups (*P* < 0.01) and consistent with our results (haplotype frequency of 19.33%) (see Table S1 and Fig. S1 at https://doi.org/10.6084/m9.figshare.7034456). There was no evidence supporting either directional or balancing selection (see Fig. S2 and S3 at https://doi.org/10.6084/m9.figshare.7034456).

### eQTL analysis reveals a role for *CREM*.

To identity possible mechanistic effects on disease susceptibility due to genetic variation in the *CUL2-CREM-CCNY* region, we compared significant SNPs with known expression quantitative trait loci (*cis*-eQTLs) identified by the Genotype-Tissue Expression (GTEx) Consortium (18). While known eQTLs overlapping with loci present in the meta-analysis of this region included representation of all three genes (*CREM*, *CUL2*, and *CCNY*), the vast majority of eQTLs that overlapped with susceptibility variants were within *CREM* (Fig. 2). Of the 254 overlapping *cis*-eQTLs for *CREM* in this region, 108 were associated within the meta-analysis (*P  < *10^−5^). In contrast, only one of the *CUL2* (*n*  =  68) and none of the *CCNY* (*n*  =  119) eQTLs overlapped with an associated GWAS SNP (*P  < *10^−5^). This suggests that the GWAS-associated SNPs may be related to *CREM* expression, and not *CUL2* or *CCNY* expression. The majority of *CUL2* eQTLs were within *CCNY* and, therefore, outside the area of the association. All *CREM* eQTLs were in a single cell type: Epstein-Barr virus (EBV)-transformed lymphocytes. Of the SNPs with overlap between the GWAS and GTEx eQTLs, the strongest effect on *CREM* expression was at rs12248333 (intronic within *CUL2*). The minor allele (G) has a MAF of 35.5%, and the presence of each allele was associated with increased odds of diarrhea-associated E. histolytica (OR_meta_ = 1.86, *P*_meta_ = 24 × 10^−6^) and decreased expression of *CREM* (effect size = −0.37, *P* value from GTEx [*P_GTEx_*] = 3.6 × 10^−6^). Of note, all overlapping eQTLs were found to decrease *CREM* expression, suggesting that SNPs in this region may impact susceptibility via decreased expression of *CREM*.

A conditional analysis was conducted controlling for a top *CREM* eQTL (rs12248333). After adjustment, the top SNP (rs58000832) decreased from an *P*_meta_ of 6.05 × 10^−9^ to an adjusted *P*_meta_ (*P*_meta,adjusted_) of 4.91 × 10^−4^ (see Fig. S4 at https://doi.org/10.6084/m9.figshare.7034456). This nonindependence between the *CREM* eQTL and the GWAS association further implicates the role of *CREM*, and not *CUL2*, in susceptibility to E. histolytica-associated diarrhea.

### E. histolytica activates cAMP signal transduction and induction of CRE-driven promoter elements via CREM.

The cAMP response element modulator (CREM) is part of a family of transcriptional regulators that regulate genes via cAMP response elements (CREs) in promoters. CREM binds conserved CREs (TGACGTCA) in regulatory regions of target genes via a basic leucine zipper DNA binding domain. Activating isoforms of CREM are regulated posttranslationally via phosphorylation. Protein kinase C (PKC) and PKA phosphorylation occurs in response to increased adenylyl cyclase activity, resulting in elevated cytoplasmic cAMP. Calcium/calmodulin-dependent kinases (CaMKs) can also phosphorylate CREM in response to T cell receptor (TCR) activation and increased calcium influx. Phosphorylated CREM transits to the nucleus, where it regulates genes via CRE binding (19).

CREM activation is transient and is repressed by the CREM isoform known as ICER (inducible cAMP early repressor). ICER is transcribed from an alternative, intronic promoter in the 3′ end of the CREM gene and lacks the kinase-inducible and *trans*-activation domains. ICER acts as a powerful inducible repressor by competing for occupancy at CREs. ICER is thought to be responsible for the transient nature of cAMP-induced gene expression. CREM and ICER isoforms and the target genes regulated are highly tissue and cell type specific (19).

**FIG 3:**
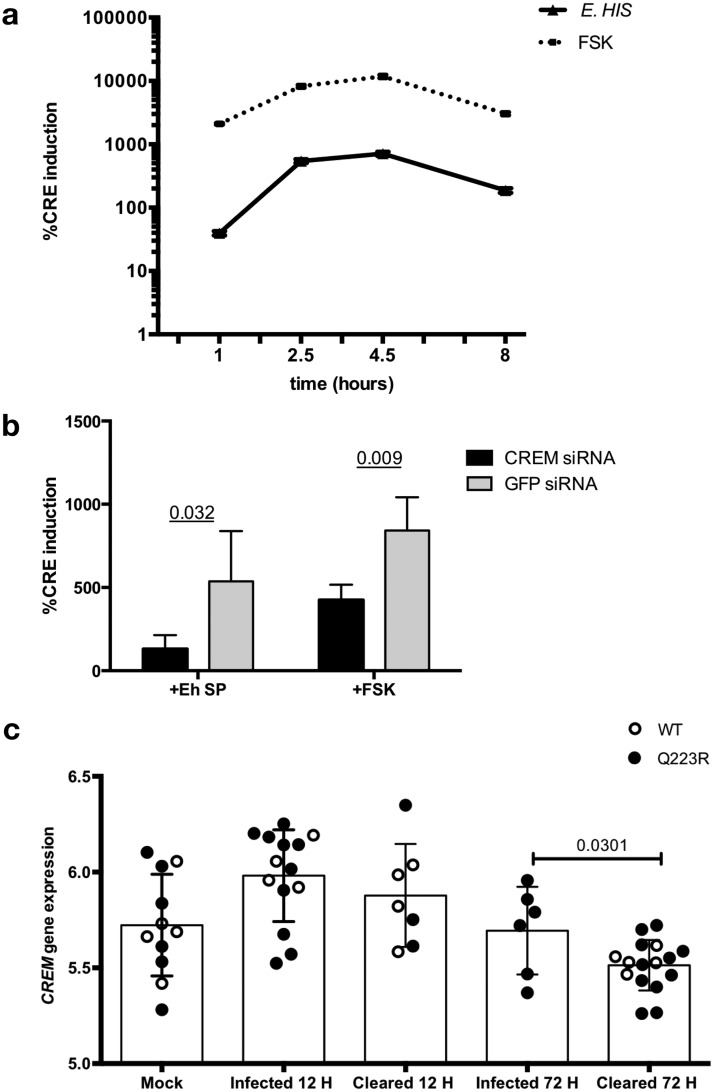
CREM is activated by E. histolytica secreted factors, and CREM expression correlates with E. histolytica clearance in mice. (a) E. histolytica activates CRE-driven gene transcription in intestinal epithelial cells. HCT116 intestinal epithelial cells transfected with a cAMP response element (CRE)-driven reporter exposed to E. histolytica trophozoites (*E*.*HIS*) or 10 μm forskolin (FSK) as a positive control for CRE activation. Five biological replicates per condition were assayed. The mean and standard errors of percent CRE induction are shown. (b) Silencing CREM decreases CRE reporter induction by soluble E. histolytica, indicating that CREM is the major transcriptional regulator acting at CREs in response to amebic secreted products. CREM was silenced by 96% compared to GFP controls by quantitative PCR (qPCR). Three biological replicates per condition were assayed. The means plus standard errors (error bars) of percent CRE induction are shown. *P* values were calculated by unpaired test. Eh SP, *E. histolytica* secreted product. (c) CREM expression is induced in early infection in mice. Mice with a wild-type humanized leptin receptor gene (WT) or susceptible leptin receptor (Q223R) were infected with E. histolytica trophozoites by intracecal injection and sacrified at 12 h or 72 h postinfection. E. histolytica infection was measured by culture of cecal contents. CREM gene expression data from microarray analysis are shown. (Data from Mackey-Lawrence et al. [12]).

Previous work has shown that amebic lysates induce significant cAMP elevation in rat colonic mucosa (20) and that secretory products of the parasite increase cAMP in leukocytes (21). We hypothesized that CREM is activated by parasite-induced cAMP signal transduction. To investigate this hypothesis, we analyzed transcriptional activation of conserved cAMP response elements by E. histolytica in intestinal epithelial cells expressing a CRE-luciferase reporter. E. histolytica induced robust CRE activation after 1 h (39.55-fold ± 3.07-fold induction), which reached ∼700-fold after 4.5 h (709.2-fold ± 53.94-fold induction). CRE activation and repression by E. histolytica and the positive-control forskolin displayed similar kinetics (Fig. 3a). To determine the contribution of CREM to E. histolytica CRE activation, we measured CRE activity in HCT116 cells silenced for CREM. Cells silenced for CREM had reduced CRE activation in response to E. histolytica secreted products (24.1% of control; *P  = * 0.032) and to the positive-control forskolin (50.3% of control; *P  = * 0.009) (Fig. 3b). These data suggest that activation of CREM drives a transcriptional response to E. histolytica via activation of CRE promoters in HCT116 intestinal epithelial cells.

### Expression of *CREM* during amebiasis.

To elucidate how *CREM* impacts susceptibility *in vivo*, we compared *CREM* expression in wild-type mice that are naturally resistant to E. histolytica and mice with increased susceptibility due to a single amino acid substitution (Q223R) in the leptin receptor. Mice were sacrificed at 12 and 72 h postinfection. Fifty percent (4/8) of wild-type mice were infected 12 h postinfection, and none were infected 72 h postinfection (0/5 infected); in contrast, 77% (10/13) of the susceptible Q223R mice were infected 12 h postinfection, and 35% (6/17) remained infected 72 h postinfection (12). *CREM* expression was increased in both susceptible and wild-type resistant mice 12 h postinfection regardless of parasite clearance. After 72 h, *CREM* expression was significantly lower in mice that had cleared the infection (both wild type and susceptible) relative to mice that were still infected (Q223R only) (−0.18 ± 0.08; *P  = * 0.031) (Fig. 3c). These data are consistent with *in vitro* data that CREM is activated early in infection and is repressed as the parasite infection is cleared.

### *CREM* knockout mice were more susceptible to amebic colitis.

**FIG 4:**
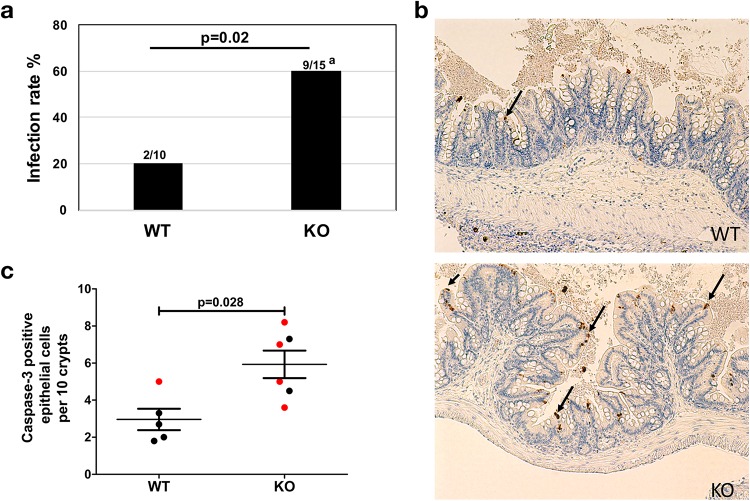
CREM^−/−^ mice (knockout [KO]) are more susceptible to amebic colitis. (a) Mice were infected with E. histolytica and euthanized on the basis of clinical score or at 72 h postinfection. Infection was assessed by the presence of live amebae upon culturing of cecal contents in growth medium. The numbers above each bar indicate the number of culture-positive mice to the total number of mice in that group. Data shown are from two independent but identical experiments. The superscript a for the KO value indicates that two mice in this group died before the 72-h time point. (b) Fixed cecal tissue was stained using antibody against active caspase-3, a marker for apoptosis (dark brown staining indicated by arrows). Representative images are shown. (c) Apoptotic death of cecal intestinal epithelial cells was scored by investigators blind to treatment groups (red circles indicate culture-positive samples). Error bars represent standard errors of the means (SEM). CREM^−/−^ mice from Blendy et al. (22) lack DNA binding domain 1a-1b and are CREM and ICER deficient.

To further determine the role of *CREM in vivo*, we compared the ability of *CREM* knockout (KO) (C57BL/6J CREM^−/−^) and wild-type (WT) mice to clear E. histolytica trophozoites introduced by intracecal injection. CREM knockout mice were more susceptible to amebic infection than wild-type mice, as assessed by culturing of cecal contents (*P  = * 0.02) (Fig. 4a). A hallmark of E. histolytica pathogenesis is potent cytotoxicity to host cells via induction of caspase-3-dependent apoptosis (23). Apoptotic death of cecal intestinal epithelial cells, as detected by caspase-3 staining, was significantly higher within the CREM^−/−^ mice (*P  =  *0.028) compared to WT mice at 72 h (Fig. 4a and c). Uninfected *CREM* KO and WT mice showed similar levels of caspase-3 staining (see Fig. S5 and S6 at https://doi.org/10.6084/m9.figshare.7034456). Higher levels of epithelial apoptosis are associated with decreased barrier function and increased inflammatory responses, both of which may contribute to more severe amebic infection in mice lacking CREM.

## DISCUSSION

The most important discovery of this work is that genetic variants in the *CREM*/*CUL2* locus are associated with Entamoeba histolytica diarrhea. Many of our most associated SNPs are also reported to be associated with inflammatory bowel disease susceptibility (24). The *CREM*/*CUL2* region was identified through the first GWAS conducted on an enteric infectious disease. Haplotype analysis and comparison with 1000 Genomes Project data suggest that this particular association is enhanced in South Asian and European populations. Expression quantitative trait loci (eQTL) analyses identified decreased *CREM* and likely not *CUL2* to be associated with amebiasis. Consistent with eQTL data, *CREM* expression was increased in mice during E. histolytica infection, and CREM^−/−^ mice had heightened susceptibility to amebic colitis.

The role of CREM as a cAMP-mediated transcriptional regulator promises to add to the understanding of intestinal health by delineating a common mechanism of gut inflammation and repair from infectious (amebiasis) and noninfectious (Crohn’s disease and ulcerative colitis) insults. The fact that a polymorphism in *CREM* underlies susceptibility to both indicates that they likely share a pathway of cAMP-dependent gene regulation, likely from an upset of the homeostatic balance of the gut microbiota with mucosal immunity.

The GWAS meta-analysis was performed on diarrhea-associated Entamoeba histolytica infection, or amebiasis, examining two separate birth cohorts: the Dhaka Birth Cohort (DBC) and the Performance of Rotavirus and Oral Polio Vaccines in Developing Countries (PROVIDE) study. These studies gave a unique opportunity to study the genetic susceptibility to enteric infection, with active surveillance capturing the majority of pediatric illness within the first year of life for these children in Dhaka, Bangladesh. The GWAS results identified a significant association (*P*_meta_ < 5 × 10^−8^) with a SNP on chromosome 10 within a region encompassing the genes *CUL2* (cullin 2) and *CREM* (cAMP-responsive element modulator). Each additional risk allele at this locus conferred a drastic 2.42-fold increased odds of E. histolytica-associated diarrhea within the first year of life. While there is high linkage disequilibrium within this region leading to an associated block of sites, the numerous known eQTLs for *CREM* overlapped with our signals, while only one of the known eQTLs for *CUL2* exhibited association with amebiasis. Functional validation showed a relationship between infection with E. histolytica and increased expression of CREM. When applied to a mouse model, CREM^−/−^ mice showed increased susceptibility to amebic colitis compared to wild-type mice. This evidence reinforces the role of *CREM* in symptomatic E. histolytica infection.

The *CREM-CUL2* region has previously been implicated in genome-wide association studies of other traits, most notably of Crohn’s disease (CD) and ulcerative colitis (UC), two major forms of inflammatory bowel disease (IBD) (24–28). Five of these loci overlap between our meta-analysis and previous studies (rs11010067, rs34779708, rs12261843, rs12242110, and rs17582416) (Table 3). Among SNPs with risk allele information, the direction of effect for IBD, Crohn’s disease, or ulcerative colitis was in the same direction of effect for amebiasis. The effect sizes were stronger for amebiasis than for IBD, with rs11010067 conferring 1.14 times the odds of Crohn’s disease within Europeans while conferring 1.75 times the odds of amebiasis within the Bangladeshi cohort (*P*_meta_ = 3.35 × 10^−5^), suggesting overlap between IBD and E. histolytica infection in this region. A conditional analysis of the peak IBD-associated SNP (rs34779708) decreased the association of our top E. histolytica variant from 6.05 × 10^−9^ to 2.89 × 10^−4^, indicating a shared role (see Fig. S4 at https://doi.org/10.6084/m9.figshare.7034456). This relationship is also confirmed in the clinical literature. It has previously been observed that the gross findings of amebic colitis can resemble those seen in inflammatory bowel disease, in which amebic colitis patients can be mistakenly diagnosed as UC or CD (7, 29). The acute stage of amebic colitis especially mimics the first attack of colonic Crohn’s disease (30).

**TABLE 3:** Relationship of known genome-wide significant associated SNPs for inflammatory bowel disease with E. histolytica diarrhea

SNP	Functional class	Disease (*n*)^*a*^	Allele	*P*	OR^*b*^	*P*_EH_^*c*^	OR_EH_^*d*^
rs11010067	Downstream gene variant	CD (25)	G	1 × 10^−26^	1.14	3.35 × 10^−5^	1.75
rs11010067	Downstream gene variant	IBD (24)	G	2 × 10^−25^	1.12	3.35 × 10^−5^	1.75
rs34779708	Intron (*CREM*)	IBD (25)	G	2 × 10^−25^	NR	9.04 × 10^−6^	1.81
rs12261843	Intron (*CCNY*)	UC (26)	G	7 × 10^−7^	1.07	2.17 × 10^−4^	1.66
rs12242110	Upstream (*CREM*)	CD (27)	G	1 × 10^−9^	1.15	2.22 × 10^−4^	1.67
rs17582416	Intergenic	CD (23)	G	2 × 10^−9^	1.16	3.40 × 10^−5^	1.75

a CD, Crohn’s disease; IBD, inflammatory bowel disease; UC, ulcerative colitis.

b OR, odds ratio; NR, not reported.

c *P*_EH_, *P* value for E. histolytica.

d OR_EH_, odds ratio for E. histolytica.

Additionally, a recent publication identified credible sets of SNPs associated with IBD in European populations (31). Of the 201 SNPs listed as the credible set for the *CREM*/*CUL2*/*CCNY* region, 186 overlap with our results and have *P  < *5 × 10^−5^, indicating a possibly shared locus (see Fig. S7 at https://doi.org/10.6084/m9.figshare.7034456). The most associated SNP within the credible set was rs4934716 (*P*_meta_ = 3.67 × 10^−6^, OR = 1.86). The linkage disequilibrium (LD) patterns between these two SNPs differ by population as measured within the 1000 Genomes Project, with higher LD within South Asian populations (*r*^2^ = 0.46) compared to European populations (*r*^2^ = 0.28). Future application of these methods in diverse populations may be able to narrow down the credible set to a likely causal SNP and be more applicable to populations like these Bangladeshi children.

These results suggest that there may be a shared pathway for pathogenesis of infection for amebiasis and for Crohn’s disease, i.e., a dysregulation in the immune response to commensal gut organisms, that includes *CREM*. The genetic relationship between infectious and inflammatory diseases is not unprecedented, as leprosy and Crohn’s disease are also known to share similar genes (32). The implications of this finding are intriguing especially since the risk allele or haplotype is common in South Asian and European populations.

The relationship between the chronic diseases characterized by aberrant mucosal response, E. histolytica-related diarrhea, and *CREM* may be along the T_h_17 pathway. A transcriptional repressor isoform of *CREM* is denoted *ICER* (inducible cAMP early repressor) and is regulated through a promoter located in one of *CREM*’s introns. Naive *ICER*/*CREM*-deficient CD4^+^ T cells have impaired functionality to differentiate to T_h_17 cells; however, this can be rescued by forced overexpression of *ICER* specifically (33). This relationship is consistent with the role of T_h_17 cells within autoimmune and inflammatory diseases. Specifically, it was found that *ICER*/*CREM*-deficient B6.lpr mice are protected from developing autoimmunity. In addition, high levels of T_h_17 have been found in the gut of subjects with Crohn’s disease (34). Mouse models have also shown that intracecal amebic infection resulted in the upregulation of T_h_17 cytokine responses to the detriment of T_h_1 cytokines (35). Further work is needed to elucidate this relationship.

There are several limitations to our analysis. We did not replicate the previously identified association with the leptin receptor despite adequate statistical power, likely due to a different case definition and study design from the original discovery analysis (11). The previous study (11) looked at all infections (asymptomatic and diarrheal) with E. histolytica within children through the preschool years, while our analysis examined only diarrhea-associated infections within the first year of life. Additional limitations may be overcome in future work, such as suboptimal power due to the small sample sizes of both studies added to our constraints in evaluating the role of copathogens. However, because the effect estimates are strong (odds ratio of 2.42 for the most associated risk allele), the combined meta-analysis allowed us to elucidate genome-wide significant associations.

In conclusion, through a meta-analysis of two separate birth cohorts, we uncovered genome-wide significant associations with diarrhea-associated E. histolytica infection in Bangladeshi infants. The top association was on a haplotype spanning *CUL2* and *CREM* on chromosome 10, a region that has previously been implicated with inflammatory bowel disease. Functional evidence suggests a role for *CREM* during early infection with E. histolytica, and *CREM* knockout mice were more susceptible to amebic colitis. The relationship between infection with the amebic parasite E. histolytica within the developing world and the development of chronic intestinal disorders in the developed world warrant further research to understand their parallels and to expand their respective treatment options.

## MATERIALS AND METHODS

The study protocol was approved by the Institutional Review Boards of the International Center for Diarrheal Disease Research, Bangladesh, University of Virginia, and Johns Hopkins Bloomberg School of Public Health. The parents or guardians of all individuals provided informed consent.

### Dhaka Birth Cohort study design.

The Dhaka Birth Cohort (DBC) (9) was part of a longitudinal birth cohort recruited from the urban slum in Mirpur Thana in Dhaka, Bangladesh, to study the influence of malnutrition in child development. A total of 629 children were enrolled within the first week after birth, beginning in January 2008, and monitored twice a week with household visits for the first year of life. Trained field research assistants took anthropometric measurements at the time of enrollment and every 3 months thereafter. The height-for-age Z-score (HAZ) and the weight-for-age Z-score (WAZ) were calculated by comparing the height and weight of the study participants with the World Health Organization (WHO) reference population, standardized for age and sex, using the WHO Anthro software, version 3.0.1. Diarrheal stool samples were collected from the home or the study field clinic every time the mother reported diarrhea. These samples were then transported to the Centre for Diarrheal Disease Research, Bangladesh (ICDDR,B) parasitology laboratory, maintaining a cold chain. The presence of Entamoeba histolytica was determined using real-time PCR (RT-PCR), as well as enzyme-linked immunosorbent assay (ELISA). A nested case-control design was utilized in which children were defined as a “case” if they had at least one diarrheal sample that was positive for E. histolytica within the first year of life by either method. Children were defined as “controls” if they had no diarrheal samples that were positive for E. histolytica within the first year of life by either method and also had at least one diarrheal or monthly stool sample tested for a true negative control.

### Dhaka Birth Cohort genotyping.

The samples from DBC were genotyped as part of a larger set of 1,573 samples from four Bangladesh study groups. These samples were genotyped in three separate batches at the University of Virginia Center for Public Health Genomics Laboratory. Sample preparation and genotype calling followed standard Illumina protocols. Only the 484 DBC samples genotyped across three batches were used for this analysis: 165 samples were genotyped in batch 1 (Illumina Human1M-duoV3; 1,199,187 total single nucleotide polymorphisms [SNPs]/copy number variations [CNV] sites); 154 samples in batch 2 (Illumina HumanOmni1-Quad v1.0; 1,140,419 total SNPs/CNV probes); and 165 samples in batch 3 (Illumina HumanOmni2.5-4v1; 2,450,000 total SNPs/CNV probes). Samples were dropped from the analysis for any of the following reasons. (i) The genotyping call rate was <95%. (ii) The samples were cryptic duplicates with differing phenotype records or were cryptically related up to the first degree in the same study group (relationships inferred with KING [36]). (iii) The inferred sex from the genetic X/Y chromosome data did not match study database gender. SNPs were dropped from analysis if any one of the following was true. (i) The per-batch call rate was <95%. (ii) The per-batch *P* value for test of Hardy-Weinberg proportions was less than 1 × 10^−4^ (X chromosome [X chr] females only). (iii) The SNPs were identified as CNV probes. (iv) SNPs mapped to multiple locations in the genome. We remapped all SNPs to Human Genome Build 37 and merged individual batches of data into a single combined data set with 529,893 common intersecting SNPs in the three batches by rs identifier (rsID). The full data set was imputed to the 1000 Genomes Phase 3 reference data (17) with phasing through SHAPEIT (37, 38) and imputation with IMPUTE2 (39–43) (see Fig. S8 to S10 at https://doi.org/10.6084/m9.figshare.7034456).

### PROVIDE study design.

The “Performance of Rotavirus and Oral Polio Vaccines in Developing Countries” (PROVIDE) Study is a randomized controlled clinical trial birth cohort that was designed to evaluate factors that may influence oral vaccine efficacy among children from areas with high poverty, urban overcrowding, and poor sanitation (15). A total of 700 children and their mothers were monitored for the child’s first 2 years of life with a 2 × 2 factorial design looking specifically at the efficacy of two-dose Rotarix oral rotavirus vaccine and oral polio vaccine (OPV) with an inactivated polio vaccine (IPV) boost. This study was performed with the International Center for Diarrheal Disease Research, Bangladesh (ICDDR,B) with the study population all from the Mirpur area of Dhaka, Bangladesh. Pregnant mothers were recruited from the community by female Bangladeshi field research assistants (FRAs). Participants had 15 scheduled follow-up clinic visits, with biweekly diarrhea surveillance at their homes by FRAs. The presence of E. histolytica in the diarrheal samples was determined by RT-PCR. Again, a nested case-control design was utilized in which children were defined as a “case" if they had at least one diarrheal sample that was positive for E. histolytica within the first year of life. Children were defined as “controls” if they had at least one diarrheal sample available for testing, but no samples were positive for E. histolytica.

### PROVIDE genetic data.

Within PROVIDE, 541 children were genotyped on Illumina’s Infinium Multiethnic Global Array (MEGA). Standard quality control metrics were used for the genome-wide data. Single nucleotide polymorphism filters included genotype missingness <5% (none), minor allele frequency (MAF) >0.5% (*M* = 659,171), and Hardy-Weinberg equilibrium *P* value of >10^−5^ (*M* = 789) (*M* indicates the number of SNPs/variants). Individuals were filtered for individual missingness <2% (none), heterozygosity outliers with F > 0.3 (*n* = 4), principal components outliers (none). One individual from each first and second degree relative pairs were removed (*n* = 36). After both individual and SNP-level filters, there were 699,246 SNPs and 499 individuals. The genetic data were split into chromosomes for phasing and imputation. Each chromosome was phased using SHAPEIT (37, 38) v2.r790 with 1000 Genomes Project Phase 3 data as the reference (17). After phasing, the chromosomes were imputed using IMPUTE v2.3.2 (39–43) using 1000 Genomes Project Phase 3 data as reference (see Fig. S8, S11, and S12 at https://doi.org/10.6084/m9.figshare.7034456).

### Cross-study genetic data harmonization.

After imputation, both data sets (Dhaka Birth Cohort and PROVIDE) were double checked for relatedness both within study, as well as between studies, to ensure independence. One individual from each pair of relateds (up to second degree relatives) was dropped (PI_HAT > 0.2). This led to 70 individuals being dropped from the Dhaka Birth Cohort and 5 from PROVIDE. Of the individuals with phenotype information, 49 samples were dropped from DBC, and 3 samples were dropped from PROVIDE. Principal-component analysis was reimplemented in PLINK (44) to determine possible heterogeneity between studies (see Fig. S13 to S15 at https://doi.org/10.6084/m9.figshare.7034456). Population substructure was found with respect to outcome in DBC and PROVIDE (see Table S3 at https://doi.org/10.6084/m9.figshare.7034456) and batch effects in DBC (see Fig. S16 at https://doi.org/10.6084/m9.figshare.7034456), and therefore batch was included as a covariate for all analyses in DBC and the first five principal components were included for both DBC and PROVIDE. Additionally, heterozygosity was calculated both within each study individually, as well as a combined data set. No outliers were found (see Fig. S17 at https://doi.org/10.6084/m9.figshare.7034456). To ensure the robustness of the imputation, we examined the cluster plots for the top genotyped SNP within the region of interest in both DBC and PROVIDE (see Fig. S18 to S20 at https://doi.org/10.6084/m9.figshare.7034456). Within DBC, the top genotyped SNPs were rs2148483 (*P*_meta_ = 9.64 × 10^−8^, OR = 2.24; *P*_DBC_ = 4.89 × 10^−4^, OR_meta_ = 2.31) and rs11595640 (*P*_meta_ = 9.25 × 10^−8^, OR_meta_ = 2.24; *P*_DBC_ = 4.89 × 10^−4^, OR_DBC_ = 2.31). The top genotyped SNPs within PROVIDE were rs7070384 (*P*_meta_ = 2.96 × 10^−7^, OR_meta_ = 2.23; *P*_PROVIDE_ = 1.77 × 10^−4^, OR_PROVIDE_ = 2.15) and rs2148483 (*P*_meta_ = 9.64 × 10^−8^, OR = 2.24; *P*_PROVIDE_ = 5.36 × 10^−5^, OR_PROVIDE_ = 2.20). All of the genotyped SNP signals were consistent with the top imputed SNPs, and the cluster plots were well separated, decreasing the chance of a false-positive result. Because of these quality control checks, we are confident in the robustness of the imputation for our top results.

### E. histolytica detection protocol.

The detection protocol for E. histolytica has previously been described by Haque et al. (45). Primers and TaqMan probes for E. histolytica (GenBank accession number X64142) were designed on a small subunit rRNA gene, with the amplified targets of 134 bp. All primers and TaqMan probes were purchased from Eurogentec (Seraing, Belgium). Multiplex real-time PCR was conducted using standard protocol (45).

### Association analyses.

To estimate the associations between genetics and diarrhea-associated *E*. *histolytica* infection, each study (DBC and PROVIDE) was initially run separately (data not shown). Logistic regression was run with SNPTEST (39, 43, 46), incorporating the imputed genotypes’ weights and assuming an additive model of inheritance. The DBC analysis was adjusted for batch and the first five principal components, while the PROVIDE analysis was adjusted for the first five principal components. HAZ at 12 months and sex were not included in any model, as they were not found to be associated with outcome in either study. The two studies were incorporated into a meta-analysis using the software META (47) in a fixed-effect analysis. Results were then filtered for MAF > 5%, info score from IMPUTE2 (INFO) > 0.6 in both cohorts, and a *P* value for heterogeneity between the two cohorts greater than 0.05. This ensured stable estimates of association that were adequately powered in both analyses separately and together. These filters resulted in a total of 6,703,908 sites assessed for association, of which genomic inflation factor (λ_GC_) = 1.023 (see Fig. S23 at https://doi.org/10.6084/m9.figshare.7034456). The per-study levels of genomic control for the sites jointly analyzed were λ_GC_ = 0.938 for DBC and λ_GC_0 = 0.931 for PROVIDE (see Fig. S24 and S25 at https://doi.org/10.6084/m9.figshare.7034456). No remarkable inflation was observed for either cohort separately or together. Conditional analyses were conducted adjusting for the additive genotypes of the top GTEx SNP (rs12248333) and IBD-associated SNP (rs34779708) with the same methods and thresholds. Permutations were conducted to assess the robustness of results. Each study was analyzed separately on “best guess” genotype calls using adaptive permutations within PLINK, with a maximum number of one million permutations. Results were then combined in a fixed-effect meta-analysis. The results were found to be robust, with the top SNP remaining highly associated (*P*  = 7.65 × 10^−8^) (see Fig. S26 at https://doi.org/10.6084/m9.figshare.7034456).

### eQTL analysis.

Known expression quantitative trait loci (eQTLs) from the Genotype-Tissue Expression project (GTEx) portal (www.gtexportal.org) were included for analysis, using association results from analyses for all tissues for *CREM*, *CUL2*, and *CCNY* (18). The overlap between these association results and the imputed meta-analysis results were included as follows: *CREM* (Epstein-Barr virus [EBV]-transformed lymphocytes), 254 sites; *CUL2* (whole blood cells-transformed fibroblasts), 68 sites, *CCNY* (esophagus-mucosa), 120 sites. Variants were determined to be both eQTLs and associated with amebiasis if the association with expression had *P*  < 10^−5^, as well as association with diarrhea-associated *E*. *histolytica* infection had *P*  < 10^−5^.

### Haplotype analyses.

For all haplotype analyses, only the genotyped original PROVIDE data were used to avoid potential bias resulting from imputation. The region of association on chromosome 10 between 35.25 Mb and 35.55 Mb was a subset of the previously phased data, including both *CREM* and *CUL2*. A total of 25 SNPs were included in this one large haplotype block. Logistic regressions assuming both additive and dominant models were used to estimate the association of the index haplotype with outcome, adjusting for the first five principal components (see Table S1 at https://doi.org/10.6084/m9.figshare.7034456). The top associated haplotype was then compared against reference populations within the 1000 Genomes Project (TGP) (17). For each continental-level population (Africa, Americas, East Asia, Europe, and South Asia), the 25 SNPs were a subset of previously phased data. There were 19 unique haplotypes found within TGP that occurred at least 10 times (on 10 separate chromosomes) across all of TGP. Enrichment for a haplotype at a continental level was assessed using a chi-squared test. Enrichment or depletion was determined per population and haplotype compared to the expected representation for that population (see Fig. S1 at https://doi.org/10.6084/m9.figshare.7034456). Haplotype associations within PROVIDE were also conducted on each gene separately based on genic coordinates (see Table S2 at https://doi.org/10.6084/m9.figshare.7034456).

### Selection analyses.

The 1000 Genomes Project data for chromosome 10 was assessed for evidence of selection using four representative populations: Bengalis in Bangladesh, Western Europeans in the United States, Han Chinese in Beijing China, and Yoruba in Nigeria. These four populations were selected to be representative of the different continents, as well as the source population for both DBC and PROVIDE. To determine the presence of positive selection, the integrated haplotype score (iHS) was calculated using selscan (48–52). To assess the presence of balancing or directional selection, Tajima’s D was calculated within vcftools (53).

### Credible set analysis.

The list of SNPs for the credible set of the *CREM*/*CUL2*/*CCNY* region associated with inflammatory bowel disease (IBD) was taken from Table S2 of Huang et al. (31). SNPs were matched on rsID and *P* value of <0.01 in the meta-analysis described in this article for amebiasis. An overlap was found for 186 of the 201 SNPs, indicating a shared locus. Linkage disequilibrium between the top credible set SNP (rs12248333) and our GWAS SNP (rs58000832) were calculated using 1000 Genomes Project reference data, specifically the European and South Asian populations.

### E. histolytica culture.

E. histolytica trophozoites were maintained in a trypsin-yeast extract-iron (TYI-S-33) medium supplemented with 2% Diamond vitamins, 13% heat-inactivated bovine serum (Gemini Labs), and 100 U/ml penicillin plus 100 μg/ml streptomycin (Invitrogen) (54). Trophozoites originally derived from E. histolytica strain HM1:IMSS (ATCC) and passed sequentially through mice to maintain animal virulence were used for challenge experiments.

### CRE-Luciferase reporter assays.

HCT116 cells were obtained from ATCC prior to beginning experiments. Cells were maintained in McCoy’s medium supplemented with 10% heat-inactivated fetal bovine serum. HCT116 cells were tested for mycoplasma with the mycocheck assay (Lonza) every 3 months. The cells were discarded after 20 passages. The cells were transfected with pCRE Tluc16-DD vector (catalog no. 88247; Thermo Fisher Scientific) using Lipofectamine 2000 (catalog no. 11668019; Thermo Fisher Scientific). The pCRE Tluc16-DD vector contains an optimized minimal core promoter and five tandem repeats of the cAMP response element (CRE), a turboluciferase reporter with a dual-destabilization domain. Luciferase levels were measured using the TurboLuc luciferase one-step glow assay kit (catalog no.88263; Thermo Fisher Scientific). Time course assays were done with cells exposed to E. histolytica at a ratio of 1 parasite to 5 host cells in transwells to delay contact-dependent cytotoxicity. The data shown are representative of three independent experiments. CREM-silenced luciferase assays were done in cells cotransfected with endoribonuclease-prepared small interfering RNA (esiRNA) targeting the second DNA binding domain CREM (catalog no.EHU125161; Sigma) or esiRNA to green fluorescent protein (GFP) as a nontargeting short hairpin RNA (shRNA) control. Three biological replicates per condition were assayed. E. histolytica secreted products were obtained as previously described (55). Percent induction was calculated (mean of biological replicates from experimental condition/mean of biological replicates of medium control) × 100. The mean and standard error (SE) of biological replicates are shown. *P* values were calculated by unpaired two-sided *t* test with no correction for multiple comparisons with Prism 6.0 (Graphpad). The data shown are representative of three independent experiments.

### *CREM* expression in Q223R mice.

*P* values were calculated by unpaired *t* test with no correction for multiple comparisons using Prism 6.0 (Graphpad).

### Challenge experiments using ICER/CREM^−/−^ mice.

C57BL/6J.CREM^−/−^ mice were derived from SV129/Bl6.CREM^−/−^ mice in which the CREM DNA binding domains were replaced by a LacZ-neo fusion cassette, as originally cloned by Blendy et al. (22) These mice were crossed to C57BL/6J mice for more than nine generations (33). Infection with E. histolytica was performed on C57BL/6J.ICER/CREM^−/−^ mice and littermate wild-type controls. Both male and female mice between 7 and 18 weeks of age were used. Mice were regenotyped to validate. Since male mice homozygous for CREM^−/−^ are sterile, we maintained a heterozygous breeding colony. Trophozoites originally derived from strain HM1:IMSS (ATCC) and passed sequentially through mice to maintain animal virulence were used for infection. Mice received a cocktail of four antibiotics (1 g/liter each ampicillin, neomycin, and metronidazole; 0.5g/liter vancomycin) in drinking water for 2 weeks prior to infection. Metronidazole was omitted from the cocktail 4 days prior to challenge. Mice were challenged in a blind manner intracecally with two million trophozoites in 150 μl medium following laparotomy (56). Mice were euthanized on the basis of their clinical score or on day 3 postchallenge, whichever came first. Cecal contents were suspended in 1 ml phosphate-buffered saline (PBS), and 300 μl was used for culturing of live amoebae in TYI-S-33 broth for up to 5 days. Infection rates were analyzed using chi-square test.

### Caspase-3 immunostaining.

Mouse cecal tissue was fixed in Bouin’s solution for 24 h and washed with 70% ethanol. Paraffin-embedded cecal sections were stained at the biorepository core facility of the University of Virginia using cleaved caspase-3-specific antibody (catalog no. 9661L; Cell Signaling). The numbers of caspase-3-positive (brown) epithelial cells and crypts were scored by investigators blinded to mouse treatments. Data were analyzed using Mann-Whitney test.

### Compliance statement.

All animal studies were conducted in strict accordance with the *Guide for the Care and Use of Laboratory Animals* (57). The protocol was approved by the International Animal Care and Use Committee at the University of Virginia (protocol 4126; PHS Assurance A3245-01). All surgeries were performed under ketamine/xylazine anesthesia; analgesics and supportive care was given to facilitate the well-being of the research animals.

### Data and code availability.

All microarray data discussed in this paper were deposited into NCBI’s Gene Expression Omnibus (58) and are accessible through GEO Series accession number GSE43372 (12). Data are publically available from the NIH, via dbGAP, phs001478.v1.p1 Exploration of the Biologic Basis for Underperformance of Oral Polio and Rotavirus Vaccines in Bangladesh or by request from the authors. All analysis programs used are detailed above, but the actual code in R for each analysis is also available by request from the authors.
